# The combination of a tyrosine kinase inhibitor and blinatumomab in patients with Philadelphia chromosome–positive acute lymphoblastic leukemia or Philadelphia chromosome‐like acute lymphoblastic leukemia

**DOI:** 10.1002/cam4.70161

**Published:** 2024-09-06

**Authors:** Xiaoxia Wu, Shenqi Lu, Xinhui Zhang, Zhen Yang, Aining Sun, Depei Wu, Huifen Zhou, Miao Miao

**Affiliations:** ^1^ Jiangsu Institute of Hematology, National Clinical Research Center for Hematologic Diseases, the First Affiliated Hospital of Soochow University Suzhou China; ^2^ Collaborative Innovation Center of Hematology Soochow University Suzhou China

**Keywords:** Blinatumomab, Philadelphia chromosome–like acute lymphoblastic leukemia, Philadelphia chromosome‐positive acute lymphoblastic leukemia, tyrosine kinase inhibitor

## Abstract

Tyrosine kinase inhibitors (TKIs) have revolutionized Philadelphia chromosome‐positive (Ph+) acute lymphoblastic leukemia (ALL) treatment. The combination of blinatumomab and a TKI in the frontline setting has shown the safety and efficacy of the chemotherapy‐free treatment approach in patients with Ph + ALL. This retrospective analysis included 19 patients with Ph + ALL and Ph‐like ALL treated with the combination of blinatumomab and a TKI. Of the 14 newly diagnosed patients, the overall response, complete remission (CR), and molecular response (CMR) rates after one cycle of blinatumomab were 100% (10/10), 90% (9/10), and 57% (8/14), respectively. Of the five relapsed patients, the CR and CMR rates were 50% (2/4) and 40% (2/5). Blinatumomab in combination with TKIs is safe and effective and hence this combination therapy could be a viable therapeutic option in front‐line treatment of patients with Ph + ALL.

Philadelphia chromosome–positive (Ph+) acute lymphoblastic leukemia (ALL) comprises approximately 25% of adult ALL cases and is historically associated with a poor prognosis. The development of tyrosine kinase inhibitors (TKIs), such as imatinib, dasatinib, and ponatinib, has revolutionized the treatment of Ph + ALL. Long‐term prognosis is unfavorable with chemotherapy alone. Research findings demonstrate that combined chemotherapy with BCR‐ABL1 TKIs results in enhanced outcomes such as complete remission (CR) rates of >90% and a 5‐year overall survival (OS) rate of 50%–75%.^1^

Blinatumomab, a bispecific T‐cell engaging (BiTE) antibody construct, has shown efficacy in treating relapsed or refractory (R/R) Ph + ALL and is approved for the same indication. In a study involving 45 patients, 36% achieved CR or CR with partial hematologic recovery (CRh), with a median OS of 7.1 months. Among responders, the minimal residual disease (MRD) negativity rate was 88%, and 44% of patients were able to receive allo‐SCT.^2^

The safety and efficacy of incorporating blinatumomab in combination with TKIs, particularly ponatinib, in the frontline setting have demonstrated the feasibility of a chemotherapy‐free treatment approach in patients with Ph + ALL, which may further improve response and survival rates in this patient population.^3^, ^4^ In addition, the response to blinatumomab in patients with Ph‐like ALL was comparable with that in those without Ph‐like ALL, thereby negating the effect of Ph‐like alterations.^1^ Herein, we report a retrospective analysis of 19 patients with Ph + ALL or Ph‐like ALL who received blinatumomab plus a TKI at our institution.

Patients with newly diagnosed Ph + ALL (*n* = 13), Ph‐like ALL (*n* = 1, with RCSD1‐ABL1 fusion gene), relapsed Ph + ALL (*n* = 3), and relapsed Ph‐like ALL (*n* = 2; one patient relapsed from the newly diagnosed cohort, one with NUP214‐ABL1) were treated with the combination of blinatumomab and a TKI. Patients who received one course of chemotherapy, with or without a TKI, were enrolled into the newly diagnosed cohort. Relapse was defined by >5% lymphoblast recurrence in bone marrow aspirate unrelated to recovery/presence of extramedullary disease. Initially, blinatumomab was administered at a standard dose of 9 μg/d for 1 week, followed by 28 μg/d for 3 weeks. However, early promising results and economic reasons led to several newly diagnosed patients (*n* = 6) receiving a “half‐course” of blinatumomab with a dose of 9 ug/d for 1 week followed by 28 ug/d for 1 week. The choice of a TKI and consolidation treatment, including allogeneic hematopoietic stem cell transplantation (allo‐HSCT), was at the discretion of the patient and the treating physician. Intrathecal chemotherapy with methotrexate (15 mg) was administered to the central nervous system (CNS) as a prophylaxis treatment every 4 to 6 weeks. MRD was assessed by six‐color multiparameter flow cytometry and quantitative reverse transcription polymerase chain reaction (RT‐qPCR) for BCR‐ABL1. ABL1 mutations were evaluated in eight newly diagnosed patients at baseline and in all five relapsed patients.

Of the 14 newly diagnosed patients, 4 (29%) were in CR at enrollment, with negative MRD confirmed by flow cytometry (<1 × 10^−4^) and a positive molecular BCR‐ABL1 level. After one cycle of TKIs and blinatumomab, 9 of 10 patients (90%) evaluable for hematologic response had a CR and an MRD negativity by flow cytometry at the same time, whereas one patient (10%) had a partial response (PR). Of note, 6 (60%) of the 10 patients received a “half‐course” of blinatumomab, including the patients who achieved PR. Among the 14 evaluable patients, 8 (57%) exhibited a complete molecular response (CMR; the absence of a detectable BCR‐ABL1 transcript by RT‐qPCR with a sensitivity of 0.01%; including 3 (50%) of 6 patients who received a “half‐course” of blinatumomab and 5 (62.5%) of 8 patients who received standard blinatumomab, Table 1). One patient with Ph‐like ALL received concurrent intrathecal chemotherapy for CNS leukemic involvement at the time of enrollment.

**TABLE 1:** Baseline characteristics and summary of outcomes of newly diagnosed patients treated with blinatumomab and tyrosine kinase inhibitor.

Patient	Age, years	Disease	Other cytogenetic abnormalities	TKD, m	Prior treatment	TKI	BiTE from diagnosis, days	Status before BiTE	Course of BiTE	Response after cycle 1	Consolidations	Best response	Relapse	Allo‐SCT	Status now	OS, months
1	45	Ph + ALL	None	ND	VP	Flumatinib	15	ND	Half	CR^a^	Chemo + TKI	CR^a^	No	Yes	CR,^a^ CMR	12
2	33	Ph + ALL	14p + c,−15,19p+,?der(20)t(15;20)(q11;q11)	Neg	VP	Dasatinib	7	Blasts <10%	Sta	CR,^a^ CMR	Chemo/ BiTE + TKI	CR,^a^ CMR	No	Yes	CR,^a^ CMR	14
3	60	Ph + ALL	+der(22)t(9;22)	Neg	Dex	Imatinib	7	Blasts <10%	Half	CR,^a^ CMR	Chemo/ BiTE + TKI	CR,^a^ CMR	No	No	CR,^a^ CMR	16
4	28	Ph + ALL	−7	Neg	Dex	Flumatinib	7	ND	Sta	CR^a^	Chemo/ BiTE + TKI	CR^a^	No	Yes	CR,^a^ CMR	19
5	33	Ph + ALL	None	ND	Dex	Flumatinib	7	NR	Half	CR,^a^ CMR	Chemo/ BiTE + TKI	CR,^a^ CMR	No	No	CR,^a^ CMR	10
6	56	Ph + ALL	None	Neg	VP	Imatinib	7	NR	Half	CR^a^	Chemo + TKI	CR,^a^ CMR	No	No	CR,^a^ CMR	7
7	34	Ph + ALL	None	Neg	Dex	Imatinib	7	Blasts <10%	Half	CR,^a^ CMR	Chemo/ BiTE + TKI	CR,^a^ CMR	No	No	CR,^a^ CMR	7
8	56	Ph + ALL	−7	Neg	VP	Imatinib	7	NR	Half	PR	Chemo + TKI	CR^a^	No	No	CR^a^	4
9	27	Ph + ALL	None	Neg	Dex	Dasatinib	7	NR	Sta	CR,^a^ CMR	BiTE + TKI	CR,^a^ CMR	No	No	CR,^a^ CMR	2
10	49	Ph‐like	t(1;9)(q22;q34),7q−	ND	IVP	Dasatinib	30	NR	Sta	CR,^a^ CMR	BiTE + TKI	CR,^a^ CMR	Yes^b^	Yes	CR,^a^ CMR	28
11	16	Ph + ALL	None	ND	VP	Olverembatinib	40	CR^a^	Sta	CR,^a^ CMR	BiTE + TKI	CR,^a^ CMR	No	No	CR,^a^ CMR	12
12	18	Ph + ALL	None	ND	Dex	Olverembatinib	30	CR^a^	Sta	CR,^a^ CMR	BiTE + TKI	CR,^a^ CMR	No	No	CR,^a^ CMR	16
13	32	Ph + ALL	None	Neg	VP	Olverembatinib	35	CR^a^	Sta	CR^a^	Chemo/ BiTE + TKI	CR^a^	No	No	CR^a^	19
14	40	Ph + ALL	None	ND	IVP	Dasatinib	30	CR^a^	Sta	CR^a^	Chemo + TKI	CR^a^	No	No	CR^a^	30

Abbreviations: allo‐SCT, allogeneic stem cell transplantation; BiTE, blinatumomab; Chemo, chemotherapy regimen; Chemo/Bite, sequential chemotherapy and blinatumomab; CMR, complete molecular remission; CR, complete remission; Dex, dexamethasone; Half, a half course of Blinatumomab; IVP, idarubicin, vincristine, dexamethasone; ND, not done; Neg, negative; NR, no response; OS, overall survival; PR, partial response; Sta, standard; TKD(m), tyrosine kinase domain mutation; TKI, tyrosine kinase inhibitor.

^a^ CR with MRD negativity by flow cytometry.

^b^ Relapse due to self‐induced discontinuation of treatment for 5 months after the second cycle.

In the subsequent consolidation therapy of the newly diagnosed patients, four (28.5%) received systemic chemotherapy plus TKI only, four (28.5%) received blinatumomab plus TKI only, and six (43%) received sequential chemotherapy and blinatumomab plus TKI. The median number of cycles of blinatumomab was 2 (range, 1–7). Up to this point, except for one patient with Ph‐like ALL experiencing relapse because of self‐induced discontinuation of treatment for 5 months after the second cycle, 13 patients (93%) are ongoing hematologic response, of which 3 patients (23%) opted for allo‐HSCT, including 2 patients displaying persistent detectable BCR‐ABL1 (<0.1%) and 1 achieving a CMR. The median duration of response in the 10 patients who did not receive allo‐HSCT was 10 months (range, 1–29 months). The OS of all 14 patients was 100%, and the disease‐free survival was 91.7% at a median follow‐up of 13 months (Figure S1).

Of the five relapsed patients, three (60%) had detectable ABL1 mutations: one had E255K and F317L simultaneously (treated with olverembatinib), one had E255K and G250E simultaneously (treated with ponatinib) and one had E255V (treated with ponatinib). The remaining two patients with negative ABL1 mutations were treated with olverembatinib and dasatinib respectively. A total of 4 (80%) patients were evaluable for hematologic response, with one patient in CRh at the time of enrollment. Patients completed a median of one cycle (range, 1–3 cycles) of blinatumomab plus a TKI, after which two patients had CR, one patient with CNS involvement had PR as the best response but relapsed during the third cycle, and one patient who relapsed from the newly diagnosed cohort with CNS leukemic involvement at the time of enrollment had a CMR of marrow but with a progressed extramedullary involvement of skin during the treatment and received systemic chemotherapy subsequently. After cycle 1, three of five patients (60%) achieved MRD negativity by flow cytometry, and two patients (40%) reached CMR (Table S1). In subsequent therapy, two patients with CR and one patient with extramedullary skin involvement received allo‐HSCT, 1 patient who relapsed during the 3rd cycle received CAR‐T cell therapy and died due to multiple‐organ dysfunction, and 1 patient with CR who did not receive allo‐HSCT had an ongoing hematological response for 11 months. After a median follow‐up of 19 months, four patients were still alive, three of them had a CMR and one patient had a positive molecular BCR‐ABL1 level after allo‐HSCT for 6 months.

During cycle 1, overall, the most frequent grade 3 or 4 adverse events (AEs) observed were thrombocytopenia (*n* = 9), neutropenia (*n* = 6), and anemia (*n* = 7). Other grade 1 to 2 AEs included cytokine release syndrome (CRS; *n* = 7), hypertension (*n* = 1), and pneumonia (*n* = 1). No serious AEs led to treatment interruptions.

In the GIMEMA LAL2116 D‐ALBA trial,^3^ 60% of 63 patients with newly diagnosed Ph + ALL who were treated with the combination of dasatinib and blinatumomab achieved CMR at the end of two cycles of blinatumomab. The MD Anderson Cancer Center initiated another phase 2 trial of blinatumomab and ponatinib in a cohort of 60 patients with newly diagnosed Ph + ALL,^4^, ^5^ where 67% of patients with newly diagnosed Ph + ALL had CMR after one cycle. Other studies also showed encouraging results of the combination of blinatumomab and a TKI for treating patients with newly diagnosed Ph + or Ph‐like ALL (Table 2).

**TABLE 2:** Blinatumomab plus TKI for patients with newly diagnosed Philadelphia chromosome–positive ALL or Philadelphia chromosome‐like ALL.

	TKI	*N*	Median age (range), years	Media cycle of BiTE	CR rate, %	CMR rate, % (c)	Median follow‐up, m	Allo‐SCT rate in CR1, %	Relapse rate, %	DFS rate, % (years)	OS rate, % (years)
GIMEMA LAL2116 D‐ALBA^6^	Dasatinib	63	54 (24–82)	≥2	98 (62/63)	60 (2), 93 (all)	53	39 (24/62)	15 (9/62)	74.6 (4)	80 0.7 (4)
MDACC^5^	Ponatinib	60	55 (20–83)	5	97 (38/39)	67 (1), 83 (all)	24	3 (2/59)	12 (7/59)	77 (3)	91 (3)
Blissphall Study^7^	Dasatinib	17	50 (22–87)	3	100 (17/17)	45 (all)	11.7	24 (4/17)	12 (2/17)	NA	NA
Nanfang Hospital^8^	Olverembatinib	13	58/34.5	≥2/1	100 (13/13)	100 (all)	7	62 (8/13)	8 (1/13)	87.5 (0.5)	100 (0.5)
Advani AS, et al.^9^	Dasatinib	24	73 (65–87)	NA	92 (22/24)	NA	32.4	5 (1/22)	32 (7/22)	77 (3)	87 (3)
GIMEMA ALLL2820^10^	Ponatinib	74	57 (20–80)	≥2	95 (55/58)	63 (all)	6.1	0	2 (1/55)	NA	NA
Present study	TKIs	14	34 (16–60)	2	93 (13/14)	57 (1), 64 (all)	13	21 (3/14)	8 (1/13)	91.7 (1)	100 (1)

Abbreviations: Allo‐SCT, allogeneic stem cell transplantation; BiTE, blinatumomab; c, cycle; CMR, complete molecular remission; CR, complete remission; DFS, disease‐free survival; m, month; N, number; NA, not applicable; OS, overall survival.

This report is a retrospective study of patients with newly diagnosed or relapsed Ph + ALL or Ph‐like ALL who received blinatumomab in combination with a TKI. In our cohort of newly diagnosed patients, after cycle 1, the overall response rate of 10 evaluable patients was 100%, with 9 patients (90%) achieving CR and a negative MRD by flow cytometry and 1 patient achieved PR. Furthermore, the CMR rate was 57% (8 of 14). Notably, 6 (43%) of the 14 patients in our cohort received a “half‐course” of blinatumomab with an escalated dose of 28 ug/d for 1 week, and 11 (78.5%) patients received first or second‐generation TKIs. However, the current study reported a comparable CMR rate after cycle one, with noteworthy molecular responses early in the induction therapy. However, as highlighted by Saliba et al,^11^ novel agents such as ponatinib and blinatumomab continue to be prohibitively expensive and inaccessible in many global healthcare settings. The “personalized approaches” elucidated in our study may expand the eligibility of many patients and increase response and OS with minimal side effects and affordable cost.

In accordance with the findings of Aldoss et al,^12^ a history of extramedullary disease was identified as a predictor of inferior response to blinatumomab therapy. Both patients in our study, with an inferior response to blinatumomab, had extramedullary involvement, of whom one patient changed the treatment to systemic chemotherapy and CD19 CAR‐T cell therapy followed by allo‐HSCT and had a persistent CR with a follow‐up of 19 months. Regrettably, the other patient succumbed to multiple organ failure after CAR‐T. Addressing this patient population necessitates the exploration of novel and effective therapies. A potential avenue involves higher dosing of blinatumomab beyond the standard dose used in ALL or the combination of blinatumomab with novel agents.^12^

This report is limited by the small sample size of the studied cohort and its retrospective nature. However, the encouraging results of the combination of blinatumomab and a TKI for treating patients with newly diagnosed Ph + or Ph‐like ALL provide proof of concept regarding its safety and efficacy. Notably, the administration of a “half‐course” of blinatumomab may broaden the eligibility of the patient population with newly diagnosed Ph + ALL offering a favorable response profile with minimal side effects and affordable cost. However, further validation through extensive clinical trials with a large number of patients is warranted.

## AUTHOR CONTRIBUTIONS

**Xiaoxia Wu:** Data curation (equal); formal analysis (equal); investigation (equal); methodology (equal); validation (equal); visualization (equal); writing – original draft (equal). **Shenqi Lu:** Data curation (equal); formal analysis (equal); investigation (equal); project administration (equal); validation (equal); visualization (equal). **Xinhui Zhang:** Data curation (equal); investigation (equal); methodology (equal); visualization (equal). **Zhen Yang:** Investigation (equal); methodology (equal); visualization (equal). **Aining Sun:** Conceptualization (equal); methodology (equal). **Depei Wu:** Conceptualization (equal); methodology (equal); project administration (equal). **Huifen Zhou:** Conceptualization (equal); investigation (equal); methodology (equal); project administration (equal); visualization (equal). **Miao Miao:** Conceptualization (equal); methodology (equal); project administration (equal); resources (equal).

## FUNDING INFORMATION

This work was supported by National Key R&D Program of China (grant number: 2022YFC2502700), National Natural Science Foundation of China (grant number: 82020108003), Priority Academic Program Development of Jiangsu Higher Education Institutions (PAPD), Jiangsu Provincial Medical Innovation Center (grant number: CXZX202201), and Suzhou Science and Technology Program Project (grant number: SLT201911).

## CONFLICT OF INTEREST STATEMENT

The authors have no competing interests.

## ETHICS APPROVAL STATEMENT

The study was approved by the Ethics Committee of the First Affiliated Hospital of Soochow University, Suzhou, China.

## PATIENT CONSENT STATEMENT

All patients provided a signed informed consent form.

## PERMISSION TO REPRODUCE MATERIAL FROM OTHER SOURCES

Not applicable.

## CLINICAL TRIAL REGISTRATION

Not applicable.

## Supporting information


**Data S1:** Supporting Information.

## Data Availability

The data that support the findings of this study are available from the corresponding author upon reasonable request.
