# Supplementary material for: The combination of a tyrosine kinase inhibitor and blinatumomab in patients with Philadelphia chromosome–positive acute lymphoblastic leukemia or Philadelphia chromosome‐like acute lymphoblastic leukemia
Source: Cancer Med. 2024 Sep 6;13(17):e70161. doi: 10.1002/cam4.70161 (PMC11378354; doi:10.1002/cam4.70161)
Supplement: Supplementary file 1 — Data S1: Supporting Information. [file CAM4-13-e70161-s001.docx]

**Supplementary Table 1**. Baseline characteristics and summary of outcomes of relapsed patients treated with blinatumomab and tyrosine kinase inhibitor

| **Patient** | **Age, years** | **Disease** | **Other cytogenetic/Gene abnormalities** | **Prior SCT** | **TKD, m** | **TKI** | **Cycles** | **Best response** | **Allo-SCT** | **Status** | **DOR, months** | **OS，**  **months** |
| --- | --- | --- | --- | --- | --- | --- | --- | --- | --- | --- | --- | --- |
| 1 | 44 | Ph+ALL | None | No | E255K,F317L | Olverembatinib | 3 | PR | No | Dead | - | 4 |
| 2 | 52 | Ph+ALL | None | No | E255K,G250E | Ponatinib | 3 | CR | Yes | Alive | 19 | 20 |
| 3 | 34 | Ph-like | NUP214-ABL1 | No | None | Dasatinib | 1 | CR, CMR | No | Alive | 11 | 12 |
| 4 | 49 | Ph-like | RCSD1-ABL1 | Yes | E255V | Ponatinib | 1 | NR | Yes | Alive | - | 19 |
| 5 | 12 | Ph+ALL | del(9)(p11.2),add(19)(q13) | Yes | None | Olverembatinib | 1 | CR, CMR | Yes | Alive | 21 | 22 |

Abbreviations: allo-SCT, allogeneic stem cell transplantation; CR, complete remission; CMR, complete molecular remission; DOR, duration of remission; NR, no response; OS, overall survival; PR, partial response; TKD(m), tyrosine kinase domain mutation; TKI, tyrosine kinase inhibitor


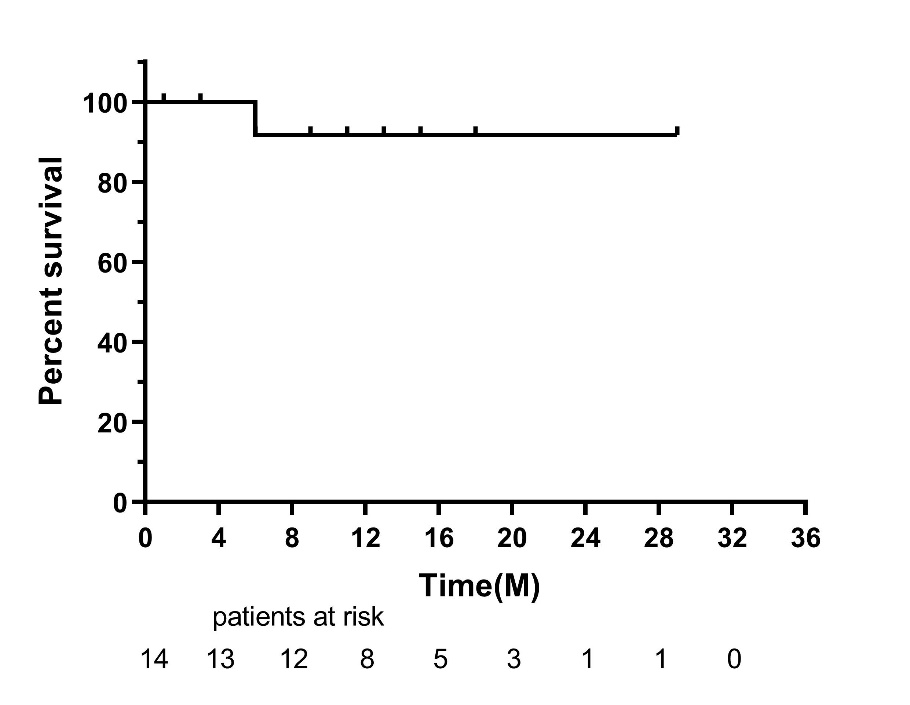


**Supplementary Figure S1**

Disease-free survival of all the newly diagnosed patients.
